# *Flt1/VEGFR1* heterozygosity causes transient embryonic edema

**DOI:** 10.1038/srep27186

**Published:** 2016-06-02

**Authors:** Yasunori Otowa, Kazumasa Moriwaki, Keigo Sano, Masanori Shirakabe, Shigenobu Yonemura, Masabumi Shibuya, Janet Rossant, Toshio Suda, Yoshihiro Kakeji, Masanori Hirashima

**Affiliations:** 1 Division of Vascular Biology, Department of Physiology and Cell Biology, Kobe University Graduate School of Medicine, 7-5-1 Kusunoki-cho, Chuo-ku, Kobe, Hyogo 650-0017, Japan; 2 Division of Gastrointestinal Surgery, Department of Surgery, Kobe University Graduate School of Medicine, 7-5-2 Kusunoki-cho, Chuo-ku, Kobe, Hyogo 650-0017, Japan; 3 Ultrastructural Research Team, RIKEN Center for Life Science Technologies, 2-3-3, Minatojima-minamimachi, Kobe, Hyogo 650-0047, Japan; 4 Institute of Physiology and Medicine, Jobu University, 270-1 Shinmachi, Takasaki, Gunma 370-1393, Japan; 5 Program in Developmental and Stem Cell Biology, The Hospital for Sick Children, Peter Gilgan Centre for Research and Learning, 686 Bay Street, Toronto, Ontario, M5G0A4 Canada; 6 Cancer Science Institute, National University of Singapore, Center for Translational Medicine, 14 Medical Drive, #12-01, 117599, Singapore; 7 International Research Center for Medical Sciences, Kumamoto University, Honjo 2-2-1, Chuo-ku, Kumamoto 860-0811, Japan

## Abstract

Vascular endothelial growth factor-A is a major player in vascular development and a potent vascular permeability factor under physiological and pathological conditions by binding to a decoy receptor Flt1 and its primary receptor Flk1. In this study, we show that *Flt1* heterozygous (*Flt1*^+/−^) mouse embryos grow up to adult without life-threatening abnormalities but exhibit a transient embryonic edema around the nuchal and back regions, which is reminiscent of increased nuchal translucency in human fetuses. Vascular permeability is enhanced and an intricate infolding of the plasma membrane and huge vesicle-like structures are seen in *Flt1*^+/−^ capillary endothelial cells. Flk1 tyrosine phosphorylation is elevated in *Flt1*^+/−^ embryos, but *Flk1* heterozygosity does not suppress embryonic edema caused by *Flt1* heterozygosity. When *Flt1* mutants are crossed with *Aspp1*^−/−^ mice which exhibit a transient embryonic edema with delayed formation and dysfunction of lymphatic vessels, only 5.7% of *Flt1*^+/−^; *Aspp1*^−/−^ mice survive, compared to expected ratio (25%). Our results demonstrate that *Flt1* heterozygosity causes a transient embryonic edema and can be a risk factor for embryonic lethality in combination with other mutations causing non-lethal vascular phenotype.

Vascular endothelial growth factor (VEGF)-A is a major player in all aspects of vascular development and is also a potent vascular permeability factor that is involved in fluid homeostasis under physiological and pathological conditions^1^,^2^,^3^. VEGF-A works through receptor tyrosine kinases expressed in endothelial cells, Flt1 and Flk1, which are also called VEGFR1 and VEGFR2, respectively^4^. Gene-targeting studies established that Flt1 serves mainly as a decoy receptor to sequester VEGF-A from activating the other primary receptor Flk1 in vascular development^5^,^6^,^7^,^8^. Interestingly, *VEGF-A* haploinsufficiency in mice resulted in embryonic lethality associated with defective blood vessel development^9^,^10^, whereas a hypermorphic allele of *VEGF-A* gene produced severe abnormalities in heart development and embryonic lethality^11^, indicating that a subtle change of VEGF-A protein levels affects vascular function during mouse embryogenesis. Although local concentration of VEGF-A protein is regulated by a decoy receptor Flt1, it has not been addressed whether *Flt1* heterozygosity affects embryonic development.

The lymphatic vascular system functions in concert with the blood vascular system to regulate the tissue fluid homeostasis of the body. Most of the extravasated interstitial fluid is absorbed back into the blood capillaries, whereas the remaining fluid and macromolecules are taken up and transported to venous circulation by lymphatic vessels. Dysfunction or impaired development of lymphatic vessels causes lymphedema^12^,^13^,^14^. We previously reported that loss of *Aspp1*, a p53-binding protein predominantly expressed in endothelial cells, causes embryonic edema with defective lymphatic development in mice^15^,^16^,^17^.

In this study, we investigated *Flt1* heterozygous (*Flt1*^+/−^) mice to determine the regulatory role of Flt1 in vascular development and fluid homeostasis since the homozygotes are embryonic lethal with a defect in early blood vessel development^5^. Here, we show that *Flt1* heterozygosity causes embryonic edema with enhanced vascular permeability. We also show that *Flt1* heterozygosity can be a risk factor for embryonic lethality in combination with other mutations causing non-lethal vascular phenotype.

## Results and Discussion

### *Flt1* ^+/−^ mice showed a transient embryonic edema without overt defects in cardiovascular development

**Figure 1:**
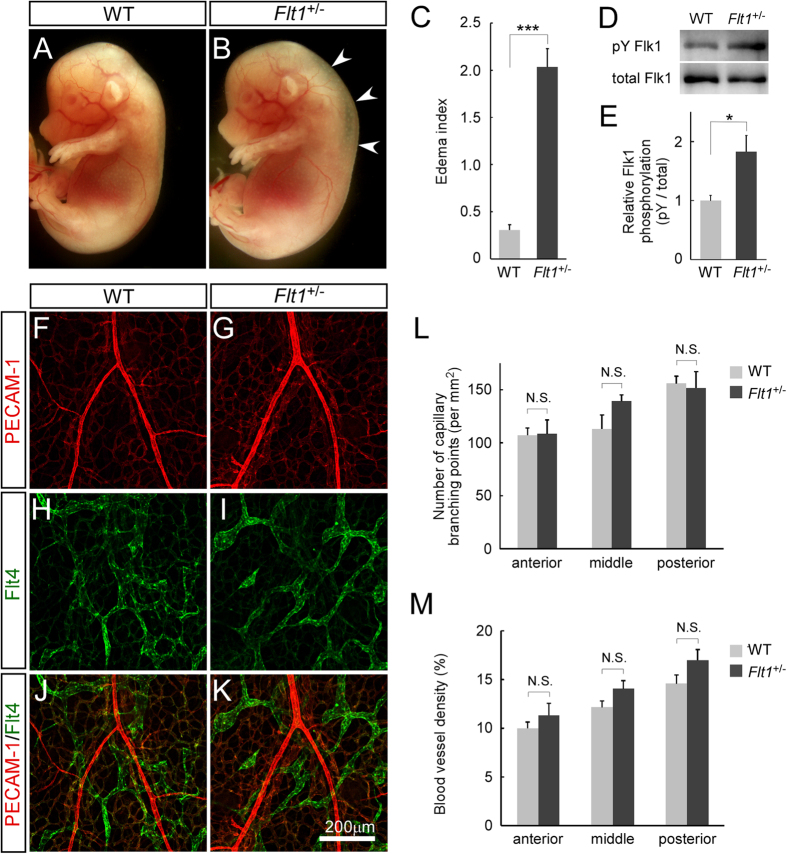
*Flt1*^+/−^ mice showed a transient embryonic edema without overt defects in vascular development. (**A**,**B**) Lateral view of whole-mount embryos at E14.5 from crosses between WT and *Flt1*^+/−^ mice. Subcutaneous edema (arrowheads) is observed only in *Flt1*^+/−^ embryos. (**C**) The edema index of *Flt1*^+/−^ embryos is significantly higher than WT embryos (WT, n = 21; *Flt1*^+/−^, n = 16). (**D**,**E**) Western blot analysis using embryonic back skin at E14.5 shows the levels of Flk1 tyrosine phosphorylation are elevated in *Flt1*^+/−^ embryos, compared to WT embryos (n = 3 each group). (**F**–**K**) Immunofluorescence confocal microscopic images of flat-mount embryonic back skin at E15.5 stained for PECAM-1 (red) and Flt4 (green). (**L**) Number of capillary branching points (per mm^2^) (n = 3 each group). (**M**) Blood vessel density (vessel area/total area of interest). There was no significant difference in blood and lymphatic vascular network between WT and *Flt1*^+/−^ embryos (n = 3 each group). Data are presented as mean ± standard error of the mean. ***P < 0.001 and *P < 0.05 as determined by unpaired two-tailed Student’s *t*-test. N.S. = not significant.

Of 263 embryos from mating wild-type (WT) female with *Flt1*^+/−^ male mice, the expected ratio of genotypes was observed at embryonic day (E) 14.5 (WT 49.0%, *Flt1*^+/−^ 51.0%). We found that *Flt1*^+/−^ embryos exhibit edema around the nuchal and back regions from E13.5 till E17.5, compared to WT embryos (Fig. 1A–C, data not shown), while edema resolves by birth. Western blot analysis using embryonic back skin shows an increased level of Flk1 tyrosine phosphorylation in *Flt1*^+/−^ embryos (Fig. 1D,E). On the other hand, when we analyzed mice harboring a *Flt1*^TK^ mutant allele lacking the tyrosine kinase domain but bearing an intact extracellular domain for VEGF-A sequestration^7^, edema was not detected in *Flt1*^+/TK^ or *Flt1*^TK/TK^ embryos (Supplementary Fig. S1). These results indicate that reduction in VEGF-A sequestration rather than Flt1 signals affects fluid homeostasis in mouse embryos.

To clarify the causes of embryonic edema in *Flt1*^+/−^ embryos, we investigated cardiovascular development. Expression analysis using mice carrying a β-galactosidase reporter knocked into the *Flt1* or *Flk1* locus and blood endothelial cells (BECs) and lymphatic endothelial cells (LECs) isolated from embryonic back skin showed that Flt1 is expressed in BECs but not in LECs, whereas Flk1 is expressed in both endothelial cell types (Supplementary Fig. S2). Whole-mount immunostaining of the back skin for a pan-endothelial marker PECAM-1 and a LEC marker Flt4 (also called VEGFR3) showed that network formation of blood and lymphatic vessels is comparable between WT and *Flt1*^+/−^ embryos at E15.5 (Fig. 1F–M and Supplementary Fig. S3). The compact layers of ventricles, trabeculation, and interventricular septum that are defective in VEGF-A-overexpressing embryos^11^ are also normal in *Flt1*^+/−^ embryos (Supplementary Fig. S4).

### *Flt1* ^+/−^ embryos showed enhancement of vascular permeability with huge vesicle-like structures in capillary endothelial cells

**Figure 2:**
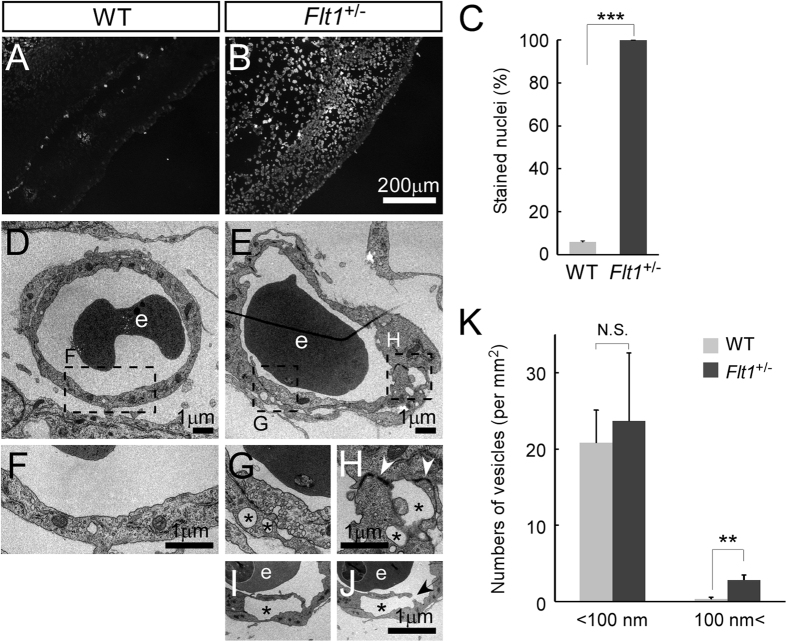
*Flt1*^+/−^ embryos showed enhancement of vascular permeability with huge vesicle-like structures in capillary endothelial cells. (**A**–**C**) Vascular permeability assay in E14.5 embryos. Frozen sagittal sections show the nuclei of BECs alone in WT embryos and those of surrounding tissues in *Flt1*^+/−^ embryos following intravenous injection of Hoechst 33258 (n = 6 each group). (**D**–**J**) Transmission electron microscopic images of capillaries in WT and *Flt1*^+/−^ embryonic back skin at E14.5. Endothelial cells of the blood capillaries containing erythrocytes (e) have an intricate intricate infolding of the plasma membrane and more huge vesicle-like structures (asterisks) which sometimes reach the lateral plasma membrane where adherens junctions reside (**H**, arrowheads) or are connected to the vascular lumen ((**I**,**J**) arrow in adjacent sections) in *Flt1*^+/−^ embryos. (**K**) Number of vesicle-like structures in capillary endothelial cells (n = 6 each group). Data are presented as mean ± standard error of the mean. ***P < 0.001 and **P < 0.01 as determined by unpaired two-tailed Student’s *t*-test. N.S. = not significant.

To look further for the causes of embryonic edema in *Flt1*^+/−^ embryos, we next investigated vascular permeability by assessing the leakage of Hoechst 33258 injected into blood vessels of embryos from crosses between WT and *Flt1*^+/−^ mice. Nuclei of BECs alone were stained in WT embryos, whereas those of tissues surrounding blood vessels were stained by Hoechst 33258 leaked out of blood vessels in *Flt1*^+/−^ embryos, indicating enhancement of vascular permeability (Fig. 2A–C). Since edema in *Flt1*^+/−^ mice is transient during mid-gestation and is not detected after birth, we performed vascular permeability in adult mice by assessing the leakage of intravenously-injected Evans blue dye after topical application of mustard oil. This analysis showed that vascular permeability is comparable between WT and *Flt1*^+/−^ adult mice (Supplementary Fig. S5), suggesting that the enhancement of vascular permeability is restricted to embryonic development in *Flt1*^+/−^ mice.

It has been shown that VEGF-A induces vascular permeability by regulating paracellular and transcellular transports in endothelial cells^18^. VEGF-A activates Src pathway leading to loosening of endothelial cell-to-cell junctions^19^,^20^,^21^,^22^, and an *in vivo* injection of VEGF-A induces the formation of vesiculo-vacuolar organelles^23^. To address the cellular basis of enhanced vascular permeability in *Flt1*^+/−^ embryos, we investigated VE-cadherin, Claudin-5, and β-catenin proteins, components of adherens junction or tight junction, but their staining patterns do not appear different between WT and *Flt1*^+/−^ embryos (Supplementary Fig. S6). Western blot analysis using embryonic back skin showed that Src activity assessed by Y416 phosphorylation (Src-pY416) is comparable between WT and *Flt1*^+/−^ embryos (Supplementary Fig. S7). Loss of pericyte coverage is also known to correlate with enhanced vascular permeability^24^,^25^,^26^, but immunostaining of a pericyte marker Desmin together with PECAM-1 showed that pericyte coverage of capillary blood vessels is not affected in *Flt1*^+/−^ embryos (Supplementary Fig. S8). Transmission electron microscopic analysis showed an intricate infolding of the plasma membrane and more huge vesicle-like structures in *Flt1*^+/−^ blood capillary endothelial cells (Fig. 2D–K). Vesicle-like structures sometimes reach the lateral plasma membrane where adherens junctions reside or are connected to the vascular lumen in *Flt1*^+/−^ embryos (Fig. 2H–J). These morphological changes in endothelial cells are most likely related to the enhanced vascular permeability in *Flt1*^+/−^ embryos although it remains to be elucidated whether paracellular and/or transcellular transports are involved.

### *Flk1* heterozygosity does not suppress embryonic edema caused by *Flt1* heterozygosity

Flk1 is the primary receptor for VEGF-A and its tyrosine phosphorylation is elevated in *Flt1*^+/−^ embryos. To investigate whether *Flk1* heterozygosity suppresses embryonic edema caused by *Flt1* heterozygosity, we crossed *Flt1*^+/−^ and *Flk1*^+/−^ mice. *Flt1*^+/−^; *Flk1*^+/−^ and *Flt1*^+/−^ embryos exhibited a comparable edema although *Flk1*^+/−^ embryos appeared normal (Supplementary Fig. S9). In addition to embryonic edema, *Flt1*^+/−^; *Flk1*^+/−^ mice surprisingly showed buphthalmia caused by abnormal aqueous humor homeostasis, as we reported previously^27^. Although *Flk1*^+/−^ mice do not exhibit embryonic edema or buphthalmia, it will be intriguing to look further into subtle vascular defects of *Flk1*^+/−^ mice in the future.

### *Flt1* heterozygosity promotes death of *Aspp1* ^−/−^ embryos

**Figure 3:**
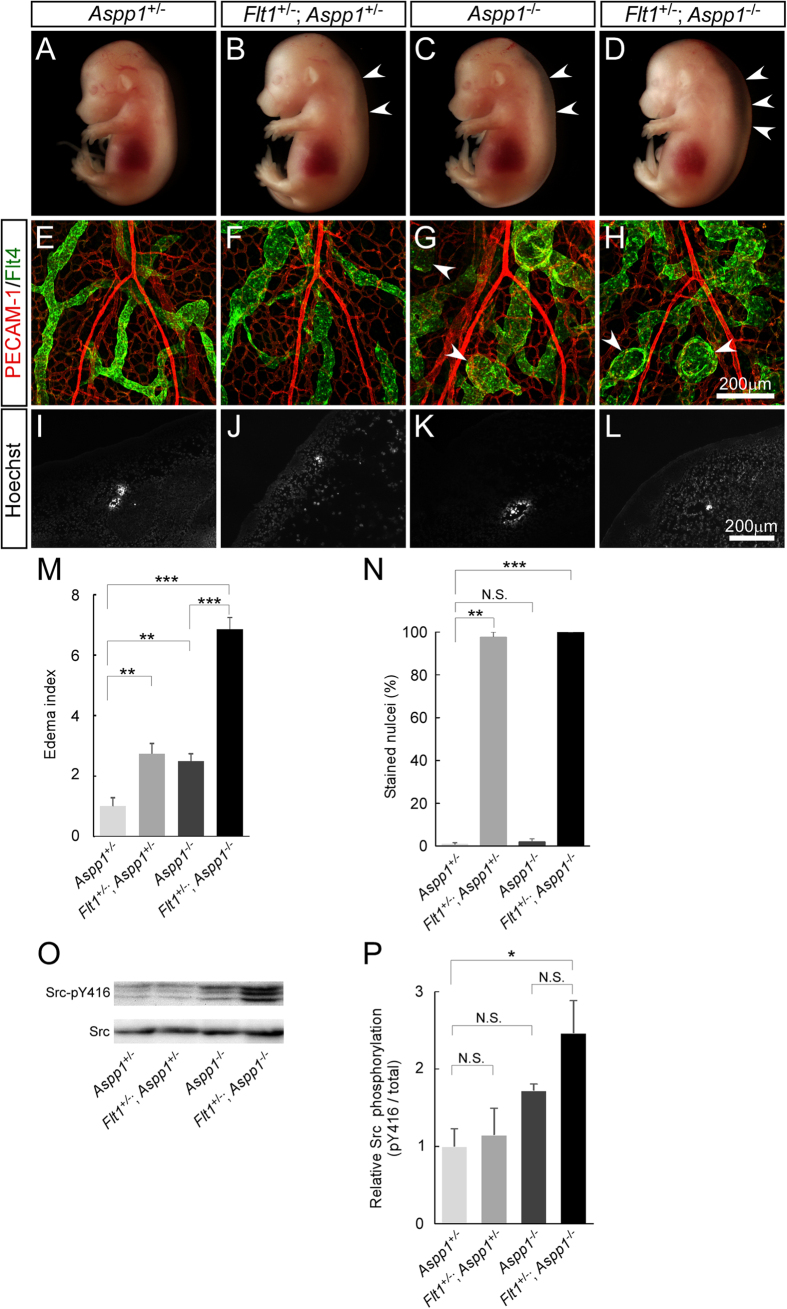
*Flt1* heterozygosity enhances embryonic edema in *Aspp1*^−/−^ embryos. (**A**–**D**) Lateral view of whole-mount embryos at E14.5 from crosses between *Flt1*^+/−^; *Aspp1*^+/−^ and *Aspp1*^−/−^ mice. Subcutaneous edema (arrowheads) is detected in all embryos except *Aspp1*^+/−^ embryos. *Flt1* heterozygosity greatly enhances edema in *Aspp1*^−/−^ embryos. (**E**–**H**) Immunofluorescence confocal microscopy images of flat-mount embryonic back skin at E15.5 stained for PECAM-1 (red) and Flt4 (green). Partly-distended lymphatic vessels and lymphatic islands (arrowheads) are detected in mice carrying *Aspp1*^−/−^ mutation. (**I**–**L**) Vascular permeability assay in E14.5 embryos. Frozen sagittal sections show the nuclei of surrounding tissues as well as BECs in mice carrying *Flt1*^+/−^ but not *Aspp1*^−/−^ mutation following intravenous injection of Hoechst 33258. (**M**) The edema index of *Flt1*^+/−^; *Aspp1*^+/−^, *Aspp1*^−/−^ and *Flt1*^+/−^; *Aspp1*^−/−^ embryos are significantly higher than *Aspp1*^+/−^ embryos (*Aspp1*^+/−^, n = 6; *Flt1*^+/−^; *Aspp1*^+/−^, n = 4; *Aspp1*^−/−^, n = 8; *Flt1*^+/−^; *Aspp1*^−/−^, n = 3). (**N**) Vascular permeability is enhanced with mice carrying *Flt1*^+/−^ mutation (n = 3 each group). (**O**,**P**) Western blot analysis using embryonic back skin at E14.5 shows that Src activity is elevated in *Flt1*^+/−^; *Aspp1*^−/−^ mice but not in other littermates (n = 3 each group). Data are presented as mean ± standard error of the mean. ***P < 0.001, **P < 0.01 and *P < 0.05 as determined by one-way analysis of variance for inter-group comparisons, and Tukey-Kramer method for group comparisons. N.S. = not significant.

We previously reported that *Aspp1*^−/−^ mice exhibit a transient embryonic edema with delayed formation and dysfunction of lymphatic vessels in embryos. The edema resolves by birth, and no overt abnormalities have been detected in adults^17^. Similarly, *Flt1*^+/−^ mice grow up normally to adult without life-threatening abnormalities, despite a transient embryonic edema caused by enhanced vascular permeability. To test whether dysregulation of fluid homeostasis may influence the viability of mouse embryos, we crossed these two mutants and analyzed occurrence and extent of edema, vascular formation, vascular permeability, and survival rate of their offspring. When we crossed *Flt1*^+/−^; *Aspp1*^+/−^ mice and *Aspp1*^−/−^ mice, all embryos except *Aspp1*^+/−^ embryos showed edema at E14.5 (Fig. 3A–D,M). *Flt1* heterozygosity greatly enhanced edema in *Aspp1*^−/−^ embryos. Moreover, only 5.7% of all offspring was *Flt1*^+/−^; *Aspp1*^−/−^ at weaning, which is significantly lower than the predicted Mendelian frequency (25%) (Table 1A). We did not notice any loss of pups during the postnatal periods between birth and weaning, suggesting that loss of *Flt1*^+/−^; *Aspp1*^−/−^ mice during embryogenesis is critical. Whole-mount immunostaining of the back skin for PECAM-1 and Flt4 showed that partly-distended lymphatic vessels and lymphatic islands, both of which are a characteristic of *Aspp1*^−/−^ embryos, are detected in mice carrying the *Aspp1*^−/−^ mutation (Fig. 3E–H). On the other hand, vascular permeability assessed by Hoechst 33258 injection is enhanced in mice carrying the *Flt1*^+/−^ mutation (Fig. 3I–L,N). When *Aspp1* mutants were crossed with *Flt1*^TK^ mutants, *Flt1*^+/TK^; *Aspp1*^−/−^ mice survived to adult at a normal Mendelian frequency (Table 1B). These results suggest that both lymphatic dysfunction and enhanced vascular permeability are responsible for stronger edema formation and lethality of *Flt1*^+/−^; *Aspp1*^−/−^ embryos. They have been reported that Src plays a role in VEGF-A-induced vascular permeability^19^,^20^,^21^,^22^ and that ASPP and Src play a cooperative role in epithelial organization in Drosophila^28^. Western blot analysis using embryonic back skin from mating between *Flt1*^+/−^; *Aspp1*^+/−^ mice and *Aspp1*^−/−^ mice showed that Src-pY416 is elevated in *Flt1*^+/−^; *Aspp1*^−/−^ mice but not in other littermates including *Flt1*^+/−^; *Aspp1*^+/−^ and *Aspp1*^−/−^ mice (Fig. 3O,P). This result suggests a synergistic regulation of Src by VEGF-A and Aspp1 although a detailed mechanism remains to be addressed.

In contrast to a reduction of Flt1 causing a transient embryonic edema, it has been reported that elevated levels of circulating soluble Flt1 are associated with preeclampsia in humans, a severe complication of pregnancy involving maternal hypertension, proteinuria and glomerular malfunction^29^. Taken together, a fine tuning of fluid homeostasis by a balance between VEGF-A and decoy receptor Flt1 is crucial for proper embryonic development and maternal health. These results also suggest that alterations in *Aspp1* or *Flt1* function could underlie the transient increase in nuchal translucency observed in some human fetuses, a defect that can still be compatible with a normal pregnancy outcome^30^,^31^. However, combined defects in pathways affecting vascular permeability and lymphatic drainage could lead to fetal loss.

## Materials and Methods

### Mice

A colony of mice heterozygous for *Flt1*^+/lacZ^, *Flk1*^+/lacZ^, *Flt1*^+/TK^, or *Aspp1*^+/lacZ^ was maintained as described previously^7^,^17^,^32^. Noon of the day on which the vaginal plug was detected was considered as E0.5. Embryos were genotyped by PCR analysis of tail DNA. Animal experiments were approved by the Institution Animal Care and Use Committee of Kobe University Graduate School of Medicine, and carried out in accordance with the animal experimentation guidelines of the Kobe University Graduate School of Medicine.

### Measurement of embryonic edema

Digital images of embryo propers were acquired with illumination from the bottom under dissecting microscope, and the number of pixels for a transparent area of embryos and crown-rump length (CRL) was measured. The extent of embryonic edema was examined as an edema index, which is a transparent area divided by the square of CRL.

### Western blot analysis

Whole embryos were dissected at E14.5, and the back skin was peeled off and lysed in 50 mmol/L Tris-HCl (pH7.4) containing 1% Triton X-100, 150 mmol/L NaCl, 10% glycerol, 1.5 mmol/L MgCl_2_, 1 mmol/L phenylmethylsulfonyl fluoride, 20 mmol/L NaF, 10 mmol/L Na_4_P_2_O7, 2 mmol/L Na_3_VO_4_, protease inhibitor cocktail, and phosphatase inhibitor. After 30 min incubation at 4 °C, the lysate was clarified by centrifugation at 10000 g for 15 min at 4 °C. The supernatant containing equal amounts of protein was used as samples for immunoprecipitation, SDS-PAGE, and western blot on PVDF membranes with antibodies to Src and Src-pY416. For detection of total and phosphorylated Flk1, Flk1 proteins were collected from the lysate by immunoprecipitation with anti-Flk1 antibody (clone 55B11), followed by western blot with antibodies to Flk1 or phospho-tyrosine (clone PY20). We used horseradish peroxidase-conjugated anti-rabbit IgG or anti-mouse IgG as secondary antibodies and enhanced chemiluminescence for detection with LAS-1000 (Fujifilm). To quantitate phosphorylated protein per total protein, the densitometric analysis of digital images was performed using the ImageJ software (National Institutes of Health).

### Immunohistochemistry

We used goat anti-Flt4, rat anti-PECAM-1 (clone Mec13.3), Armenian hamster anti-PECAM-1 (clone 2H8), rat anti-LYVE-1 (clone ALY7)^33^, rabbit anti-β-galactosidase, rat anti-VE-cadherin (clone 11D4.1), mouse anti-Claudin-5 (clone 4C3C2), rabbit anti-β-catenin, goat anti-Flk1, and mouse anti-Desmin (clone D33) antibodies. For immunofluorescence, we used Alexa Fluor 488-, Cy3-, or Cy5-conjugated secondary antibodies.

For immunohistochemical analysis of embryonic back skin, whole embryos were dissected at E14.5 and E15.5 and fixed in phosphate-buffered saline (PBS) containing 4% paraformaldehyde at 4 °C for 10 minutes. The back skin was peeled off and further fixed in the same fixative at 4 °C for 3 hours. Tissues were washed in PBS with 0.2% Triton X-100 (PBT) at 4 °C for 30 minutes twice, blocked in PBT containing 1% bovine serum albumin at room temperature for 1 hour, and stained with primary antibodies in blocking solution at 4 °C overnight. Tissues were washed in PBT for 30 minutes 3 times at 4 °C and twice at room temperature, followed by staining with secondary antibodies in blocking solution at 4 °C overnight. Tissues were washed in PBT for 30 minutes 3 times at 4 °C and twice at room temperature. Back skin was flat-mounted on slide glasses in ProLong Antifade and analyzed by confocal laser scanning microscopy (FV-1000, Olympus or LSM 700, Zeiss). Quantification of pericyte coverage was measured on the number of pixels of PECAM-1^+^ blood vessels and Desmin^+^ pericytes.

### Vascular permeability assay

Embryos were recovered with intact yolk sac and placenta at E14.5, perfused individually with Hoechst 33258 (2 μg/embryo) from the vitelline vein for 5 minutes, and dissected further to collect the embryo propers. The embryos were mounted in OCT compound and cut sagittally in 10 μm thick cryosections. The distribution of stained nuclei was examined by inverted fluorescent microscopy (IX81, Olympus). The extent of vascular permeability of dermal blood vessels was measured as the number of stained nuclei per total cell number in a circular area of 20000 μm^2^.

Adult mice at 6 to 7 weeks of age were anesthetized and injected with Evans blue dye (30 mg/kg in 300 μl) into a tail vein. After 1 minute, 5% mustard oil in mineral oil was applied to the dorsal and ventral surfaces of the ear with a cotton tip. After 30 minutes, mice were perfused with 10 ml of PBS containing 1% paraformaldehyde to wash out dye from blood vessels. Ears were removed, dried at 55 °C overnight, and weighed. Evans blue was extracted from the ears in 1 ml of formamide at 55 °C overnight and measured with spectrophotometry at OD610. The standard curve was prepared from serial dilutions of Evans blue dye at known concentration in formamide and used to determine the concentration in samples. The extent of vascular permeability was measured as the weight of extracted dye per weight of ear^34^. We analyzed one ear per mouse.

### Transmission electron microscopic analysis

Trunk of embryos was fixed in 0.1 M cacodylate buffer (pH7.4) containing 2% paraformaldehyde and 2% glutaraldehyde at room temperature for 2 hours and at 4 °C overnight. After the fixation, the samples were postfixed in 1% osmium tetroxide in the same buffer for 1 hour on ice, dehydrated in a series of graded ethanol, and embedded in resin. Thin sections were cut with an ultramicrotome (ULTRACUT, Leica) and examined in an electron microscope (JEM-1010, JEOL Ltd.).

### Reverse transcription-polymerase chain reaction (RT-PCR)

Total RNA was isolated by an RNeasy Mini Kit and RNase-Free DNase Set from PECAM-1^+^/LYVE-1^−^/CD45^−^ BECs and PECAM-1^+^/LYVE-1^+^/CD45^−^ LECs purified from the back skin at E14.5, as described previously^17^. First-strand cDNA was synthesized using the Super Script first-strand synthesis system for RT-PCR according to the manufacturer’s instructions, and used for PCR analysis with specific primers for Flt1 (5′-TGTGGAGAAACTTGGTGACCT-3′ and 5′-TGGAGAACAGCAGGACTCCTT-3′), Flk1 (5′-AGAACACCAAAAGAGAGGAACG-3′ and 5′-GCACACAGGCAGAAACCAGTAG-3′), PECAM-1 (5′-CAAACCGTATCTCCAAAGCC-3′ and 5′-TCTGTGAATGTTGCTGGGTC-3′), LYVE-1 (5’-TTCCTCGCCTCTATTTGGAC-3′ and 5′-ACGGGGTAAAATGTGGTAAC-3′), Prox1 (5′-AAGTGGTTCAGCAATTTCCG-3′ and 5′-TGACCTTGTAAATGGCCTTC-3′) and β-actin (5′-GGACTCCTATGTGGGTGACGAGG-3′ and 5′-GGGAGAGCATAGCCCTCGTAGAT-3′).

### Hematoxylin and Eosin (H&E) staining

E14.5 embryos were fixed in PBS containing 4% paraformaldehyde at 4 °C overnight, dehydrated into methanol, embedded in paraffin, cut in 5 μm thick sections, and stained with H&E by standard procedures.

### Statistical analysis

Values are presented as mean ± standard error of the mean. The unpaired two-tailed Student’s *t*-test was used to compare the results between two groups. One-way analysis of variance was used for inter-group comparisons, and Tukey-Kramer method was used for group comparisons. Chi-square test was performed to compare the actual survival to expected numbers. At least three specimens were analyzed in all cases. *P*-values are indicated as follows; ***P < 0.001, **P < 0.01, and *P < 0.05.

## Additional Information

**How to cite this article**: Otowa, Y. *et al. Flt1/VEGFR1* heterozygosity causes transient embryonic edema. *Sci. Rep.* **6**, 27186; doi: 10.1038/srep27186 (2016).

## Supplementary Material

Supplementary Information

## Figures and Tables

**Table 1:** Heterozygosity of *Flt1* knockout allele but not of *Flt1* ^TK^ allele promotes death of *Aspp1* ^−/−^ mice.

(A) Number of live mice from crosses of *Flt1*^+/−^; *Aspp1*^+/−^ and *Aspp1*^−/−^ mice							
Stage	Litter no.	*Aspp1*^+/−^	*Flt1*^+/−^; *Aspp1*^+/−^	*Aspp1*^−/−^	*Flt1*^+/−^; *Aspp1*^−/−^	Total	*P*-value
E13.5 (%)	1	7 (46.6)	3 (20.0)	4 (26.7)	1 (6.7)	15	N.S.
E14.5 (%)	12	33 (24.8)	31 (23.3)	37 (27.8)	24 (18.1)	125	N.S.
			2 (1.5)†		6 (4.5)†	8†	
E15.5 (%)	4	18 (33.3)	11 (20.4)	9 (16.6)	11 (20.4)	49	N.S.
				1 (1.9)†	4 (7.4)†	5†	
E16.5 (%)	1	1 (16.7)	3 (50.0)	0 (0)	2 (33.3)	6	N.S.
E17.5 (%)	5	11 (20.4)	19 (35.2)	14 (25.9)	3 (5.5)	47	N.S.
					7 (13.0)†	7†	
At weaning (%)	56	158 (36.2)	130 (29.8)	123 (28.2)	25 (5.8)	436	<0.001
Expected (%)		(25)	(25)	(25)	(25)		
**(B) Number of live mice from crosses of** ***Flt1***^**+/TK**^; ***Aspp1***^**+/−**^ **and** ***Aspp1***^**−/−**^ **mice**							
**Stage**	**Litter no.**	***Aspp1***^**+/−**^	***Flt1***^**+/TK**^; ***Aspp1***^**+/−**^	***Aspp1***^**−/−**^	***Flt1***^**+/TK**^; ***Aspp1***^**−/−**^	**Total**	***P*****-value**
At weaning (%)	35	109 (29.8)	83 (22.7)	80 (21.8)	94 (25.7)	366	N.S.
Expected (%)		(25)	(25)	(25)	(25)		

^†^Embryos found dead at dissection is indicated.
