# Supplementary material for: Flt1/VEGFR1 heterozygosity causes transient embryonic edema
Source: Sci Rep. 2016 Jun 2;6:27186. doi: 10.1038/srep27186 (PMC4890026; doi:10.1038/srep27186)
Supplement: Supplementary Information [file srep27186-s1.pdf]

SUPPLEMENTARY FIGURES AND FIGURE LEGENDS

Flt1/VEGFR1 heterozygosity causes transient embryonic edema

Yasunori Otowa, Kazumasa Moriwaki, Keigo Sano, Masanori Shirakabe, Shigenobu Yonemura, Masabumi Shibuya, Janet Rossant, Toshio Suda, Yoshihiro Kakeji, and Masanori Hirashima

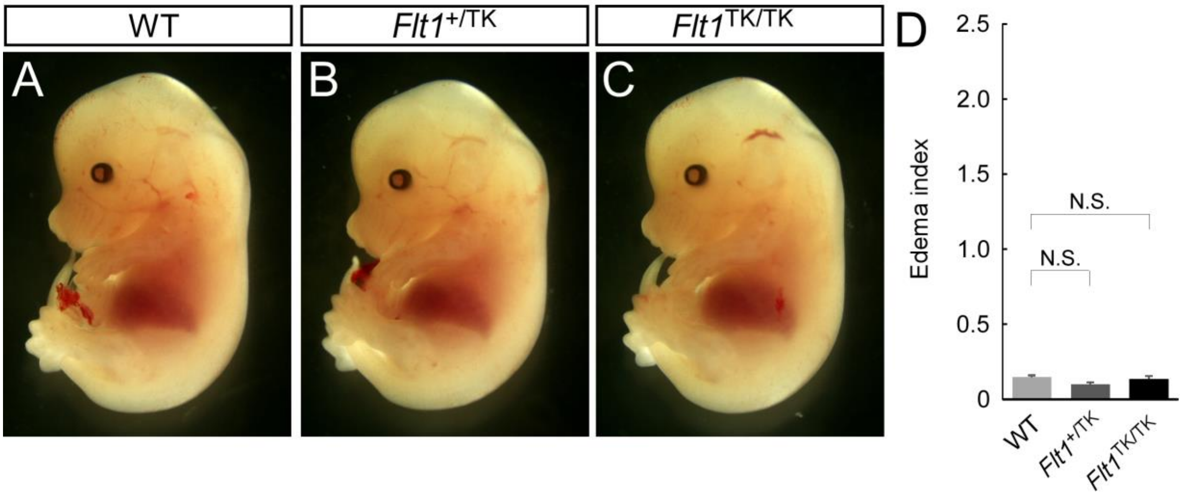

Supplementary Figure S1. *Flt1*$^{+/TK}$ mice do not show embryonic edema. (**A-C**) Lateral view of whole-mount embryos at E14.5 from crosses between *Flt1*$^{+/TK}$ and *Flt1*$^{+/TK}$ mice. (**D**) There was no difference in edema index among all genotypes (WT, n = 3; *Flt1*$^{+/TK}$, n = 3; *Flt1*$^{TK/TK}$, n = 3). Data are presented as mean ± standard error of the mean. Statistical comparison was performed by one-way analysis of variance for inter-group comparisons, and Tukey-Kramer method for group comparisons. N.S. = not significant.

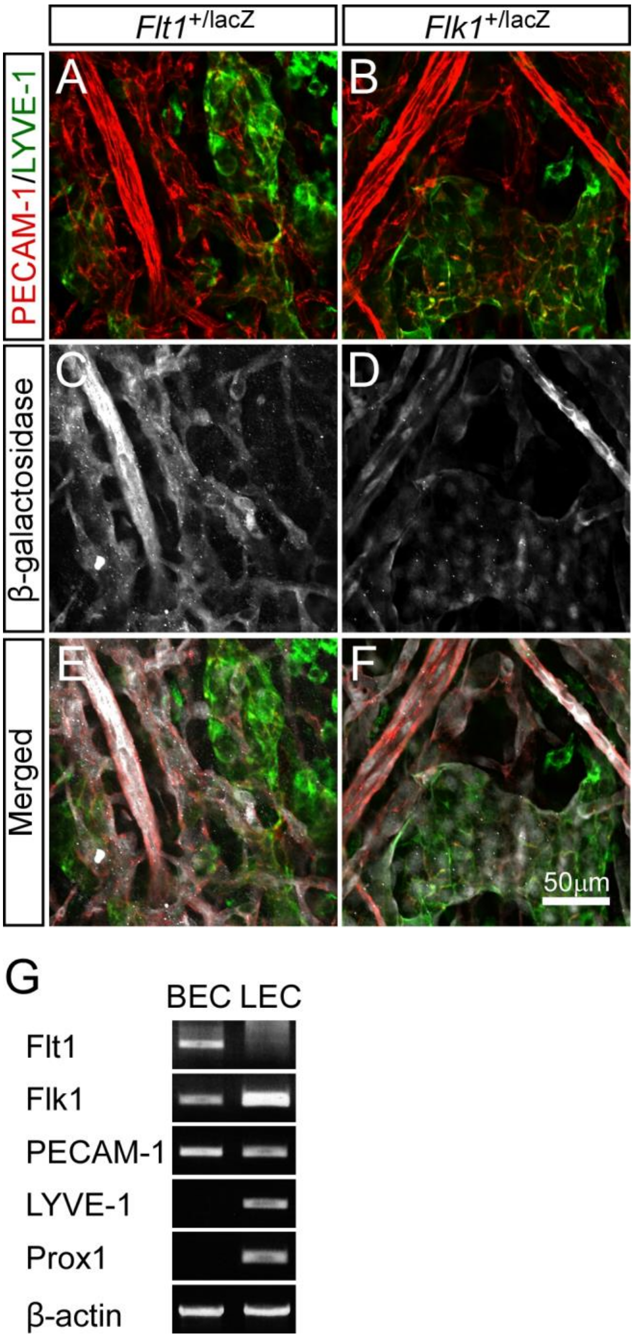

Supplementary Figure S2. Flt1 is expressed in BECs but not in LECs. (**A-F**) Immunofluorescence confocal microscopic images of flat-mount embryonic back skin at E15.5 stained for a pan-endothelial marker PECAM-1 (red) and a LEC marker LYVE-1 (green), and β-galactosidase (white), a reporter of Flt1 or Flk1 expression. Flt1 is detected in BECs but not in LECs, whereas Flk1 is detected in both endothelial cell types. (**G**) RT-PCR analysis of endothelial gene expression in BECs and LECs from embryonic back skin at E14.5.

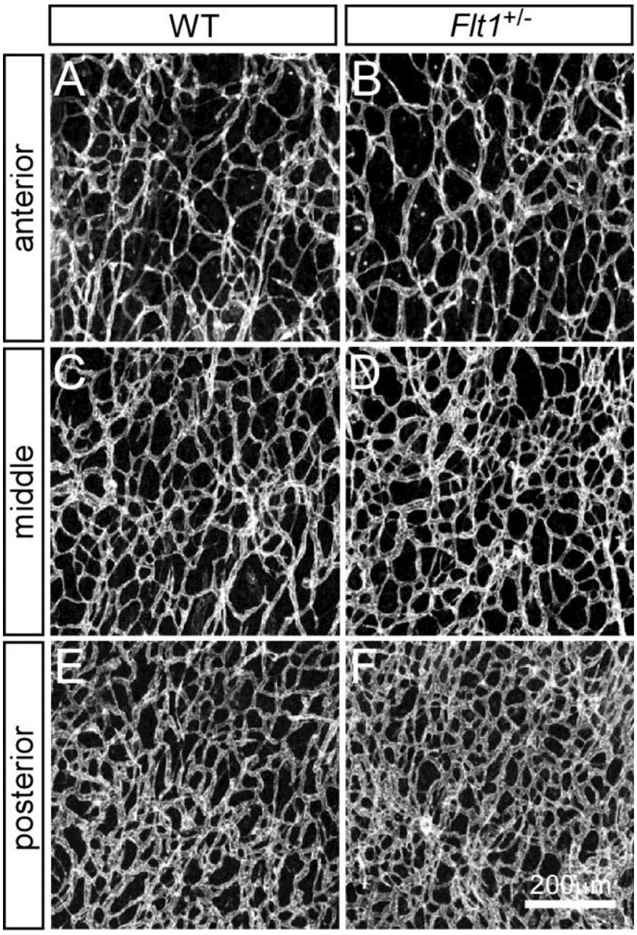

Supplementary Figure S3. Network formation of capillary blood vessels is normal in *Flt1*$^{+/-}$ embryos. (**A-F**) Immunofluorescence confocal microscopic images of flat-mount embryonic back skin around midline at E15.5 stained for PECAM-1.

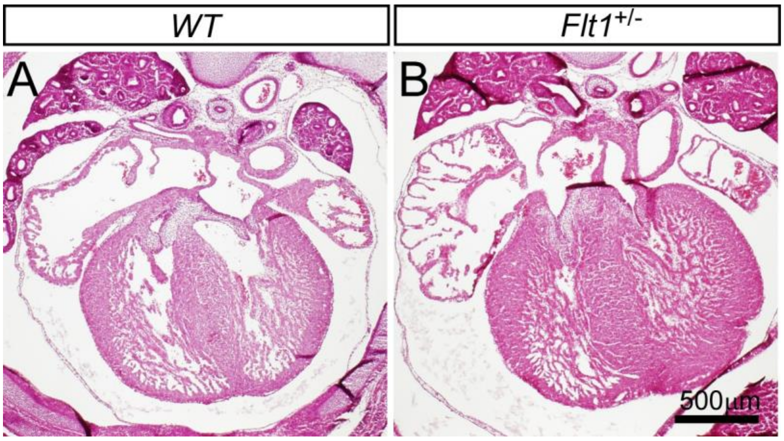

Supplementary Figure S4. Heart development is normal in *Flt1*$^{+/-}$ embryos. (**A-B**) H&E staining of cross-sections of embryonic heart at E14.5 shows that the heart is morphologically normal in *Flt1*$^{+/-}$ embryo.

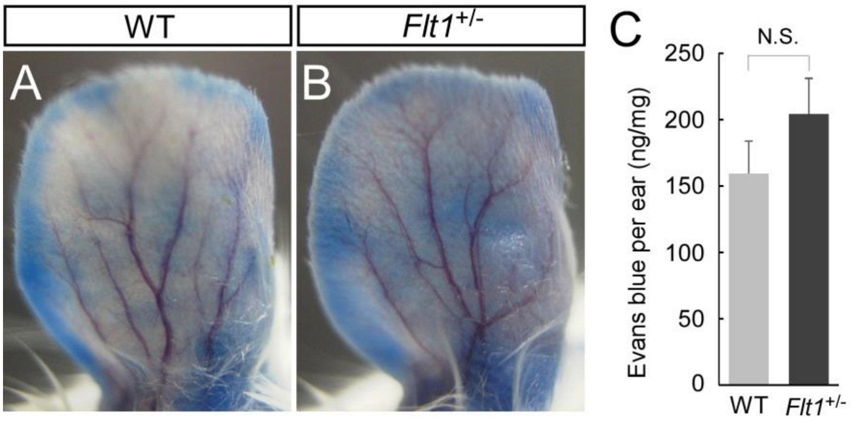

Supplementary Figure S5. Vascular permeability is not affected in *Flt1*$^{+/-}$ adult mice. (**A, B**) Images of mouse ears after injection of Evans blue dye into the tail vein. (C) The amount of extravasated Evans blue dye was measured in ear skin after treatment with inflammatory agents. There was no significant difference between WT (n = 10) and *Flt1*$^{+/-}$ (n = 11) mice. Data are presented as mean ± standard error of the mean. Statistical comparison was performed by unpaired two-tailed Student's t-test. N.S. = not significant.

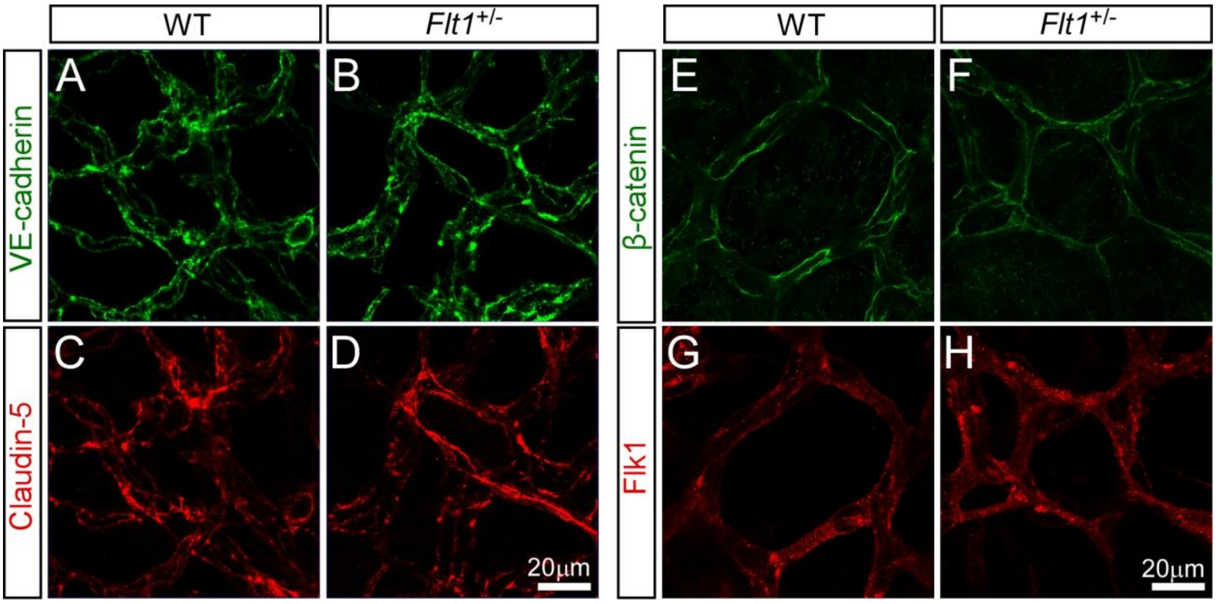

Supplementary Figure S6. Staining patterns of endothelial cell-to-cell junction molecules do not appear significantly different between WT and *Flt1*$^{+/-}$ embryos. (**A-D**) Immunofluorescence confocal microscopic images of flat-mount embryonic back skin at E14.5 stained for VE-cadherin (green) and Claudin-5 (red). Staining patterns do not appear different between WT (n = 9) and *Flt1*$^{+/-}$ (n = 9) embryos. These images are representative of n=9. (**E-H**) Immunofluorescence confocal microscopic images of flat-mount embryonic back skin at E14.5 stained for β-catenin (green) and Flk1 (red). Staining patterns do not appear significantly different between WT (n = 10) and *Flt1*$^{+/-}$ (n = 10) embryos.

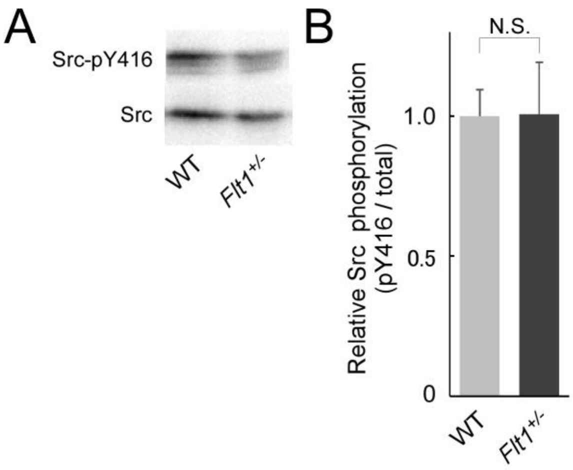

Supplementary Figure S7. Src activity is comparable between WT and *Flt1*$^{+/-}$ embryos. (**A-B**) Western blot analysis using embryonic back skin at E14.5 shows that Src activity assessed by Y416 phosphorylation (Src-pY416) is comparable between WT and *Flt1*$^{+/-}$ embryos (n = 3 each group). Data are presented as mean ± standard error of the mean. Statistical comparison was performed by unpaired two-tailed Student's *t*-test. N.S. = not significant.

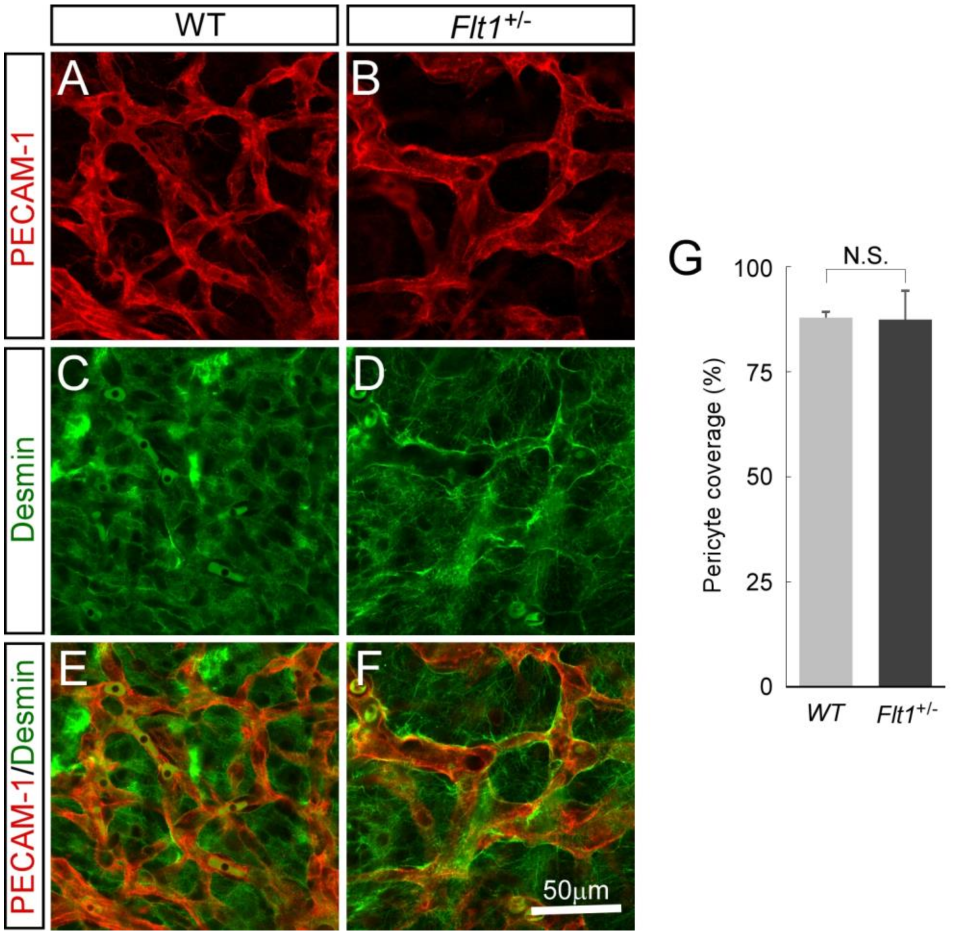

Supplementary Figure S8. Pericyte coverage is normal in *Flt1*$^{+/-}$ embryos. (**A-G**) Immunofluorescence confocal microscopic images of flat-mount embryonic back skin at E14.5 stained for PECAM-1 (red) and Desmin (green) are comparable between WT and *Flt1*$^{+/-}$ embryos (n = 3 each group). Data are presented as mean ± standard error of the mean. Statistical comparison was performed by unpaired two-tailed Student's *t*-test. N.S. = not significant.

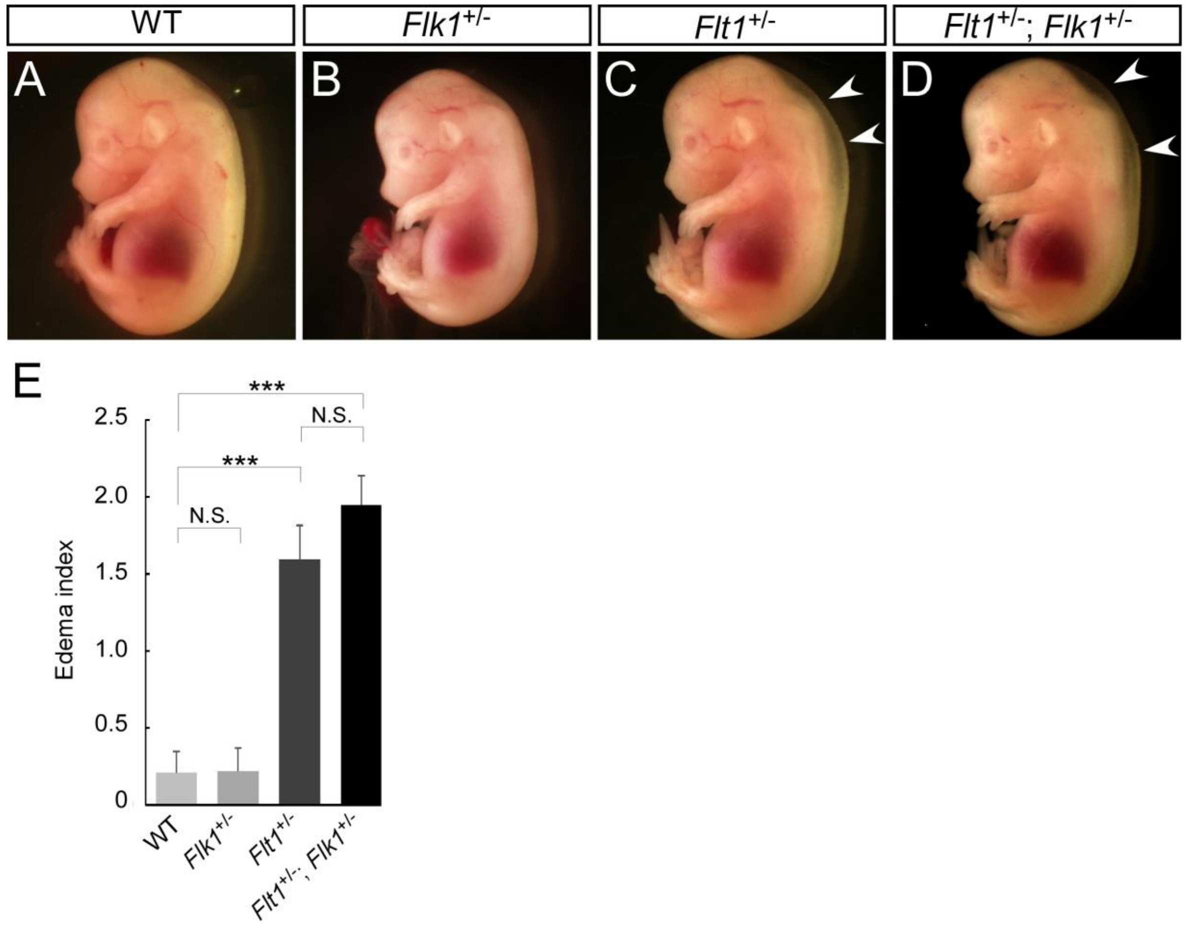

Supplementary Figure S9. *Flk1* heterozygosity does not suppress embryonic edema caused by *Flt1* heterozygosity. (**A-D**) Lateral view of whole-mount embryos at E14.5 from crosses between *Flt1*$^{+/-}$ and *Flk1*$^{+/-}$ mice. Subcutaneous edema (arrowheads) is detected in *Flt1*$^{+/-}$ embryos regardless of *Flk1* genotype. (**E**) The edema index of *Flt1*$^{+/-}$ and *Flt1*$^{+/-}$; *Flk1*$^{+/-}$ embryos is significantly higher than WT and *Flk1*$^{+/-}$ embryos (WT, n = 15; *Flk1*$^{+/-}$, n = 13; *Flt1*$^{+/-}$, n = 6; *Flt1*$^{+/-}$; *Flk1*$^{+/-}$, n = 8). Data are presented as mean ± standard error of the mean. ***P<0.001 as determined by one-way analysis of variance for inter-group comparisons, and Tukey-Kramer method for group comparisons. N.S. = not significant.
